# Influence of Substrate in Roll-to-roll Coated Nanographite Electrodes for Metal-free Supercapacitors

**DOI:** 10.1038/s41598-020-62316-0

**Published:** 2020-03-24

**Authors:** Nicklas Blomquist, Rajesh Koppolu, Christina Dahlström, Martti Toivakka, Håkan Olin

**Affiliations:** 1 Mid Sweden University, Department of Natural Sciences, Sundsvall, SE-851 70 Sweden; 2 Åbo Akademi University, Laboratory of Paper Coating and Converting, 20500 Turku, Finland; 3 Mid Sweden University, Department of Chemical Engineering, Sundsvall, SE-851 70 Sweden

**Keywords:** Supercapacitors, Chemical engineering, Mechanical engineering, Electronic properties and devices

## Abstract

Due to the high electric conductivity and large surface area of nanographites, such as graphene and graphite nanoplatlets, these materials have gained a large interest for use in energy storage devices. However, due to the thin flake geometry, the viscosity of aqueous suspensions containing these materials is high even at low solids contents. This together with the use of high viscosity bio-based binders makes it challenging to coat in a roll-to-roll process with sufficient coating thickness. Electrode materials for commercial energy storage devices are often suspended by organic solvents at high solids contents and coated onto metal foils used as current-collectors. Another interesting approach is to coat the electrode onto the separator, to enable large-scale production of flat cell stacks. Here, we demonstrate an alternative, water-based approach that utilize slot-die coating to coat aqueous nanographite suspension with nanocellulose binder onto the paper separator, and onto the current collector as reference, in aqueous metal-free supercapacitors. The results show that the difference in device equivalent series resistance (ESR) due to interfacial resistance between electrode and current collector was much lower than expected and thus similar or lower compared to other studies with a aqueous supercapacitors. This indicates that electrode coated paper separator substrates could be a promising approach and a possible route for manufacturing of low-cost, environmentally friendly and metal-free energy storage devices.

## Introduction

Due to the high electric conductivity and large surface area of nanographites, such as graphene and graphite nanoplatlets, these materials have gained a large interest for use in energy storage devices and printed electronics^1–4^. Nanographites can be produced by a large variety och routes, but for large-scale production liquid exfoliation techniques seem to be the most promising methods^5–9^. To promote low-cost, sustainable and environmentally friendly devices, water-based solvents is preferred in the process^7,9,10^ together with bio-based binders, such as cellulose, instead of polytetrafluoroethylene (PTFE) and polyvinyl alcohol (PVA)^3,4,11–14^. However, due to the thin flake geometry of nanographites the viscosity and yield stress of the suspension is high even at solids contents of a few percent. These suspension also exhibits a very low water retention which in combination with the low solids content is challenging to coat in a roll-to-roll process with sufficient coating thickness for conductive layers or energy storage electrodes. One possible route for large-scale coating of nanographite electrodes is with slot-die coating. High shear rates can be achieved in a pressure driven flow when the suspension is passed through a narrow slot, causing a reduction in its apparent viscosity (for shear-thinning suspensions). The suspension with reduced viscosity can exit the slot and be transferred immediately to the substrate and form a uniform film.^15,16^.

An important aspect in aqueous energy storage is the choice of material for the current-collector. Commercial energy storage often use aluminum and copper foil which provide excellent conductivity and simple cell assembly together with organic electrolytes. To utilize the favorable cost and environmental aspects of energy storage with aqueous electrolytes the current collectors needs to be corrosion resistant due to the aggressive nature of the aqueous environment^17,18^. Except for the well-known corrosion-resistant materials such as titanium, stainless steel, nickel and platinum, modified aluminum foil and graphite foil have shown promising results in previous studies for aqueous Li-ion batteries (LIBs) and supercapacitors (SCs)^3,17^. Electrode materials for commercial energy storage devices are often suspended by organic solvents into pastes which are coated onto metal foils used as current-collectors, followed by heat treatment and calendering to minimize the electrical resistance between electrode and current collector. Two electrode-coated current-collectors are then put together with a separator in between forming a battery or SC cell^18,19^. Another interesting approach, for large-scale electrode fabrication, is to coat directly on the separator, preferably on both sides, and then attach the current-collectors in the stage of cell assembly. This approach would also enable large-scale production of flat cell stack modules were bipolar plates are used instead of traditional current collectors^20^.

In this work we address these challenges and study the difference in electrode and device properties for electrodes coated onto the paper separator versus the contact in aqueous SCs. We utilized slot-die coating to coat nanographite suspension with nanocellulose binder onto substrates of paper and graphite foil to investigate the difference in electrode and device properties. The results shows that the electrode thickness and porosity differs between the substrates coated with identical material and with the same wet coating thickness. We could also see that the difference in device equivalent series resistance (ESR) due to interfacial resistance between electrode and current collector was much lower than expected. Compared to other studies with a aqueous SCs the ESR was similar or low for both coated substrates, indicating that electrode coated paper separator substrates could be a promising approach for use with bipolar plates for direct cell stacking of flat SC modules. The achieved results are an important step towards increased understanding on large-scale electrode fabrication from nanographite/nanocellulose composite suspensions and a possible route for manufacturing of low-cost, environmentally friendly and metal-free energy storage devices.

## Results and Discussion

### Electrode coating

**Figure 1:**
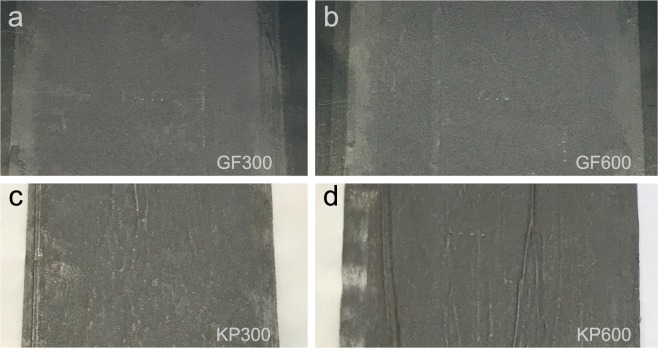
Photographs of the coated substrates where (**a**) GF300 and (**b**) GF600 are the graphite foil samples with one and two layers of 300 *μ*m wet coating respectively, and (**c**) KP300 and (**d**) KP600 are the kraft paper samples with one and two layers of 300 *μ*m wet coating respectively.

To investigate the influence of substrate in roll-to-roll coated nanographite electrodes for metal-free supercapacitors, in terms of electrode sheet resistance, SC device specific capacitance (C_*sp*_) and ESR, samples with two different substrates were prepared; graphite foil (current collector) and kraft paper (separator). To avoid clogging in the applicator and achieve a coatable suspension with a viscosity close to standard coating colors, the solids content was adjusted to 3.5%. The suspension viscosity is compared with standard coating color in the methods section. The substrates were coated with nanographite-nanocellulose suspension in a continuous roll-to-roll coater with slot-die applicator. One and two layers was applied on each substrate with 300 *μ*m wet coating thickness for each layer. All samples showed good adhesion to the substrate and resulted in fairly smooth electrodes; see Fig. 1.

Figure 1shows photographs of the coated substrates where (a) GF300 and (b) GF600 are the graphite foil samples with one and two layers of 300 *μ*m wet coating respectively, and (c) KP300 and (d) KP600 are the kraft paper samples with one and two layers of 300 *μ*m wet coating respectively. It can be seen that the coated kraft paper has some wrinkles, which is not present for the graphite foil, and this is due to swelling and shrinking of the substrate during coating and drying. One of the challenges with coating of nanographite-nanocellulose suspensions is the low solids content and low water retention, generating insufficient coat weight with traditional blade or film coating. With a slot-die applicator the wet coating thickness can be much higher but this leads to a large amount of water in the coatings and a rapid wetting of the substrate, resulting in longer drying period and a higher probability of substrate and coating wrinkles if the substrate changes dimensions.

**Figure 2:**
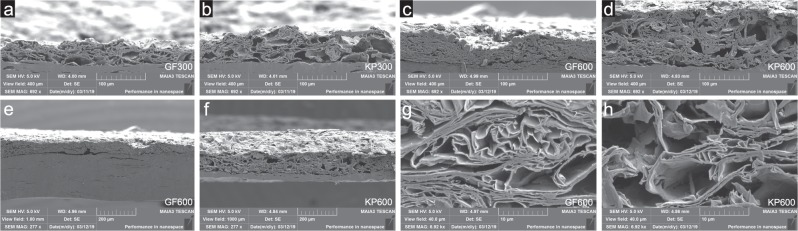
SEM cross-section images of (**a**) GF300, (**b**) KP300, (**c**) GF600 and (**d**) KP600, with a view field of 400 *μ*m in width. (**e**,**f**) Shows low magnification images of GF600 and KP600 cross-sections respectively and (**g**,**h**) shows high magnification cross-sections of the electrode in GF600 and KP600 respectively.

Figure 2 shows cross-section images of the coated substrates. Figure 2a–d shows GF300, KP300, GF600 and KP600, with a view field of 400 *μ*m in width. Figure 2e,f shows the cross-sections from GF600 and KP600 with a view field of 1000 *μ*m, and Fig. 2g,h shows cross-sections with a higher magnification of the electrodes with a 40 *μ*m wide view field.

From SEM cross-sections it can clearly be seen that there are a difference in coating thickness between the substrates, for both one and two layers of coating. All images are oriented with the substrate in the bottom (solid gray). The coating thickness varies slightly along the cross section but was approimately 50 *μ*m for GF300, 60 *μ*m for KP300, 70 *μ*m for GF600 and 110 *μ*m for KP600, which fits well with the thicknesses measured with the digital indicator. Measured electrode thickness with deviation is shown in Table 1. The measured porosity was close to 89% for all samples. In Fig. 2g,h it can be seen that the GF600 electrode is more dense (smaller pores) compared to the electrode in KP600, this is also visible in 2c–f even if the magnification is lower. The thickness difference between GF300 and KP300 is much smaller but from 2a,b its possible to see a slightly more dense electrode in GF300 compared to KP300. This is most likely an calendering effect in the last rolls of the coating setup, where the electrode faces the rolls and passes a nip before rewind. This nip was used to adjust the substrate tension (web tension) during coating. Different rates of compression is generated at this nip depending on the substrate thickness, wet coating thickness and web tension. Furthermore, The cross sectional images show that all samples has relatively large pores or cavities between clusters of nanographite flakes. This could be reduced if the electrodes were calendered after coating, to generate more contact area between the flakes and smaller pores with less unused volume but probably to the expense of ion permeability.

**Table 1:** Electrode and device properties for the coated samples.

Sample	Substrate	Wet coating thickness [*μ*m]	Dry coat weight [gm^−1^]	Electrode thickness [*μ*m]	Sheet resistance [Ω sq^−1^]	ESR [Ω]	Specific capacitance [Fg^−1^]
GF300	Graphite foil	300	10.5	43.2 ± 6.3	0.041	0.88	70.0
GF600	Graphite foil	300 + 300	21.0	67.5 ± 5.9	0.045	0.69	46.3
KP300	Paper separator	300	10.5	58.7 ± 3.9	6.762	1.12	48.3
KP600	Paper separator	300 + 300	21.0	109.2 ± 7.3	3.426	0.75	30.8

The specific capacitance is calculated from GCD at a current density of 0.52 mA/cm^2^, which corresponds to 0.5 Ag^−1^ for one layer samples and 0.25 Ag^−1^ for two layer samples. The ESR was calculated from GCD at a current density of 1.04 mA/cm^2^.

### Electrical measurements

Table 1shows the electrode and device properties for all coated samples.

The dry coat weight was calculated from the wet coating thickness and the solids content of the nanographite-nanocellulose suspension. The calculated dry coat weight was 10.5 gm^−2^ and 21.0 gm^−2^ for 300 *μ*m and 600 *μ*m of wet coating thickness respectively. Sheet resistance was measured on the electrode-coated substrates and ESR together with specific capacitance was measured on the SC-units. All units had a symmetrical set of electrodes (equal mass loading in positive and negative electrode), the ESR was calculated from galvanostatic charge-discharge measurements (GCD) at a current density of 1.04 mA/cm^2^ and the specific capacitance is calculated from GCD at a current density of 0.52 mA/cm^2^, which corresponds to 0.5 Ag^−1^ for one layer samples and 0.25 Ag^−1^ for two layer samples.

The specific capacitance for GF600 and KP300 is similar to a previous study with the same nanographite and binder, but then with an addition of 50% to 90% low-cost activated carbons with high surface area^3^. GF300 stands out in this study with a noteworthy specific capacitance in relation to the low-cost and environmentally friendly materials used. This result is similar to studies on inkjet printed graphene (48–132 Fg^−1^)^21^ and carbon nanotube coated cotton fibers (70–80 Fg^−1^)^22^ but low compared to small-scale electrodes based on functionalized graphene (135 Fg^−1^)^23^ or holey graphene flakes (170–350 Fg^−1^)^24^. However, all of these studies used more complex and thus more expensive materials and processes. The ESR was similar or low compared to other studies on aqueous SCs^25–27^, but still large for high power applications.

**Figure 3:**
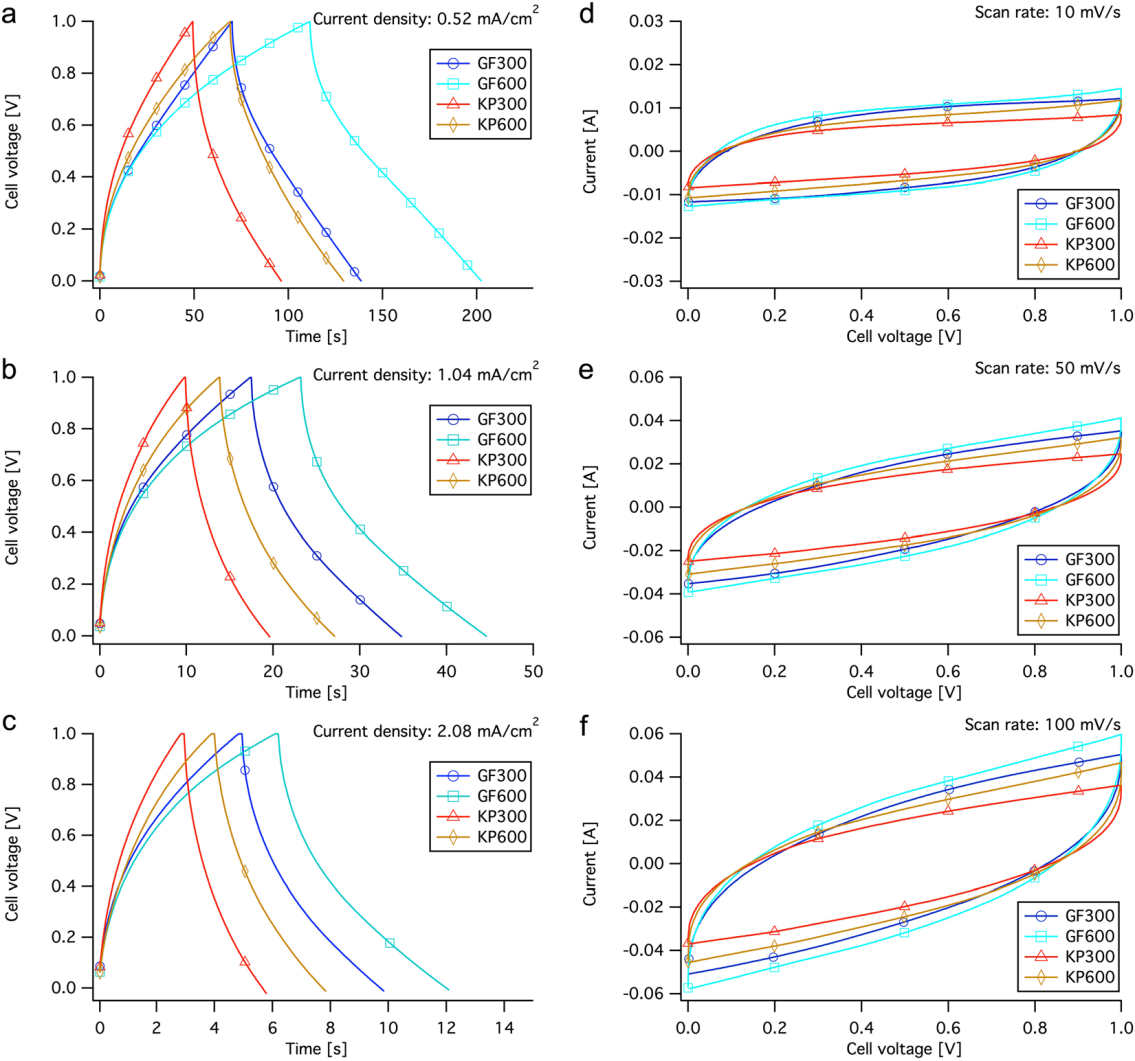
Data plots from galvanostatic charge-discharge (GCD) and cyclic voltammetry (CV) measurements of SC devices from the four different samples. Constant-current curves from GCD at charge and discharge current densities of (**a**) 0.52 mA/cm^2^, (**b**) 1.04 mA/cm^2^ and (**c**) 2.08 mA/cm^2^. I-V curves from CV with scan rates of (**d**) 10 mV/s, (**e**) 50 mV/s and (**f**) 100 mV/s. The SCs were cycled for 10 cycles at each scan rate and current density and the capacitance and ESR were obtained from the 10th cycle.

Figure 3a–c shows constant-current curves from GCD of the four units with three different charge and discharge current densities. The current densities were (a) 0.52 mA/cm^2^, (b) 1.04 mA/cm^2^ and (c) 2.08 mA/cm^2^. The result indicated that the curve shapes of all units were slightly bent at low cell voltage during charging and at high cell voltage during discharge. This can be seen as a measure of charge propagation (charge transfer) in the devices, were an ideal linear curve shape corresponds to ideal charge propagation. This behaviour can be an effect of a high interfacial resistance between electrode and current collector, partly due to cavities in the interface and partly due to the fact that the devices exhibits low compression during measurements. Since GF600 and KP600 has two layers of electrode coating they are expected to have approximately twice the charge and discharge times, and thus twice the device capacitance, compared to GF300 and KP300. It can be seen from the results that GF600 has higher capacitance than GF300 and KP600 has higher capacitance than KP300 for all current densities, but far from twice. It can also be seen that at low current density GF300 shows similar capacitance as KP600, containing twice the amount of electrode material. This can be linked to the calendering effect of the electrodes in the web tension nip. The samples with two coated layers have passed the nip once for each layer and since the porous electrodes easily can be compressed, the pores in the first layer may be partly blocked or compressed to a size not accessible for the electrolyte ions. This would result in inaccessible surface area and lower specific capacitance. The ESR is correlated to the interfacial resistance between electrode and current collector, the resistance of electrolyte and the inner resistance of ion diffusion in the electrode. ESR is used to determine the rate that the supercapacitor can be charged and discharged, and thus an important factor in high power applications. The resistive drop between charge and discharge, and thus the device ESR, is lower for GF600 and KP600 compared to the other devices and it can be seen that GF300 and GF600 has lower ESR than KP300 and KP600 when comparing devices with the same coat weight.

Figure 3d–f shows I-V curves from cyclic voltammetry measurements (CV) with three different scan rates, (d) 10 mV/s, (e) 50 mV/s and (f) 100 mV/s. The shapes of the CV measurements showed that no reactions occurred, other than electrostatic charging and discharging. The differences among the units were evident the curve shapes and the current plateaus, which can be linked to the curve shapes and resistive drop in GCD measurements in Fig. 3a–c. The different curve shapes indicated that, for KP300 and KP600, a higher cell voltage was required to obtain the same charge current density as that of GF300 and GF600 (the bending distance is longer), indicating a greater resistance to charge transfer in the electrode. The difference is most evident at a scan rate of 10 mV/s and most probably a result of the ion permeability in the electrode combined with the interfacial resistance. All devices contained two paper separators, in order to keep identical cell structure for all samples, which leads to a twice as long travel distance trough the separator than needed, thus leading to longer diffusion times. This explanation is supported by the results at higher scan rate, were the bending distance gets higher for all devices. At low scan rates, the ions have sufficient time to diffuse trough the separator and into the pores of the electrode, whereas at high rates, only a part of the ions reach the electrode and only the easily accessible pores are accessed^18,28,29^. This would also result in lower capacitance at higher current densities and high scan rates, which is evident in the decrease of discharge times compared to current density in GCD measurements along with the plot area during discharge in the CV measurements.

**Figure 4:**
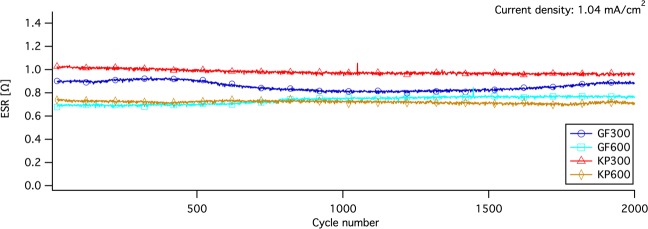
Device ESR cycle stability data over 2000 cycles of constant current cycling (0 V - 1 V - 0 V) at a current density of 1.04 mA/cm^2^ for all units.

Figure 4 shows device ESR cycle stability data over 2000 cycles of constant current cycling (0 V - 1 V - 0 V) at a current density of 1.04 mA/cm^2^ for all units. KP300 and KP600 shows stable ESR values over 2000 cycles with a small decrease over time. GF300 and GF600 are less stable, were the ESR in GF300 decreases between 500 and 1500 cycles. Both GF300 and GF600 show a small net increase in ESR after 2000 cycles. The cyclability study indicates good ESR cycle stability, despite the use of low-cost materials, aqueous electrolyte, water-based binder solution and coating suspension along with a scalable coating technique. In previous studies^3,4,9^ we have showed that high shear forces are needed to produce the nanographites and also to mix the nanographite with nanocellulose. When the composite suspension is dried, the particles in composite are held together by the nano-sized cellulose fibrils in a web-like formation forming a robust composite with good mechanical stability and wet strength. This, together with the graphite foil current collectors, can be an explanation to the good ESR cycle stability. It is also highly probable that both ESR and electrode charge propagation would be improved significantly by larger compression of the devices and hence increasing the contact area between electrode particles and between the electrode and current collector. This would however require a more advanced cell encapsulation or the use of bipolar plates to directly stack flat cells into modules.

To conclude, we demonstrated a possible large-scale route for manufacturing of nanographite-nanocellulose electrodes. With a slot-die applicator it is possible to get electrodes with sufficient coat weight for supercapacitor applications, even at a suspension solids content of 3.5%. The result showed differences in both electrode and device properties for electrodes coated onto the paper separator versus the contact in aqueous supercapacitors. The coating thicknesses was lower and more dense for the graphite foil samples compared to the samples coated on the paper separator, probably due to an calendering effect in the coating setup at the rolls before rewind. The samples with two layers of electrode coating showed much lower specific capacitance compared to samples with one layer, indicating either that the actual coat weight was lower than calculated or that a part of the material in the electrode could not be accessed by the electrolyte ions. In terms of specific capacitance, the electrode-coated graphite foil samples was superior electrode-coated paper separator. However, the difference in ESR due to interfacial resistance between electrode and current collector was much lower than expected. Furthermore, the specific capacitance for GF300, GF600 and KP300 is noteworthy in a wider perspective. Compared to commercially available electrode material and advanced new developments the specific capacitance is low, however, considering the low-cost materials used, the potential low-cost production process and the fact that the electrodes only contains nanographite and cellulose, the performance per unit cost is high.

## Methods

### Materials

The electrode materials used in this study were nanographite and nanocellulose. The nanographite was used to provide good electrical conductivity and surface area. Cellulose nanofibrils (CNF) was used as a binder to improve strength and stability in the electrodes as described by Andres *et al*.^4^. The nanographite was processed according to the method described by Blomquist *et al*.^9^ using thermally expanded graphite (EXG 9840, Graphit Kropfmühl, Germany) as the initial material. The nanographite was mixed with 10%wt CNF and the solids content of the suspension was adjusted to 3.5% (35 *gl*^−1^) by removing weather with filtration. The cellulose used was a once-dried kraft pulp supplied by SCA, Sweden. The CNF preparation is based on the method described by Saito *et al*.^30^ with some modifications. A detailed description of the procedure are described by Andres *et al*.^31^. After the chemical pretreatment, the suspension with a dry content of 2% was prepared and dispersed using an Ultra Turrax T50 and a dispersing element S50N-G45F (both from IKA-Werke GmbH & CO. KG) for 30 min at 8100 rpm to obtain the nanofibrils. Two different substrates was used; untreated grease proof paper (45 gm^−2^ kraft paper from Nordic Paper, Sweden) and graphite foil (Sigraflex F02012TH from SGL Group, Germany). 1 M Sulfuric acid was used as electrolyte for the assembled SC devices.

### Rheology

Rheology measurement of the suspension was carried out using a Paar Physica MCR300 rheometer (Anton Paar GmbH). A Couette geometry (radii of bob and cup are 13.3 and 14.5 mm respectively, vertical gap between bob and cup was kept at 5.7 mm) was used and the measurement was done according to the standards specified in DIN 53019-1. Shear flow measurement was performed with a shear rate ramp of 0.1–1000 s-1 with 20 s per data point. The viscosity unit used was milipascal seconds (mPas) Table 2 shows the viscosity of the nanographite-nanocellulose suspension together with three other suspensions, at three different shear rates for comparison.

**Table 2:** Comparison of viscosity for different suspension at three shear rate points.

Suspension	Viscosity at 10 mPas	Viscosity at 100 mPas	Viscosity at 1000 mPas
Nanographite-nanocellulose (3.5% solids)	1400	260	65
Standard coating color (CaCO_3_ 55% solids)	1200	260	80
Cellulose nanofibrils (3% solids)	4000	970	170
Cellulose nanocrystals (3% solids)	2400	400	40

### Roll-to-roll coating process

**Figure 5:**
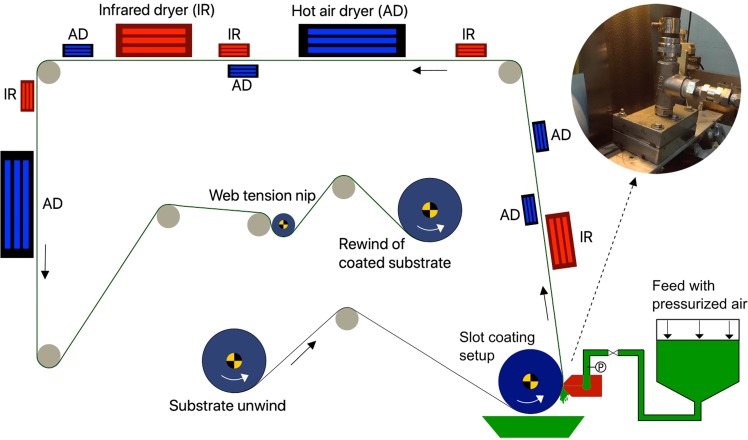
Schematic sketch of the roll-to-roll coating setup.

The substrates were coated using a Rotary Koater (RK PrintCoat Instruments Ltd.), which is a laboratory scale roll-to-roll pilot coating machine with operating speeds between 1 and 50 mmin^−1^, and depending on the requirement, it can be fitted with various coating heads. It was equipped with a 5 kW infrared drying unit and two 10 kW hot-air dryers with adjustable airflows and temperatures reaching 200 °C. The suspension was fed into a custom built slot-die (length 34 mm, width 74 mm, slot gap 500 *μ*m, and distribution channel diameter 16 mm) from an air-pressurized feed vessel. The slot-die was installed at a 3 o’clock position relative to a backing roll and was used as both coating applicator and metering device, same setup as used by Koppolu *et al*.^15^ to coat cellulose nanocrystals on pigment coated paperboard. Figure 5

shows the schematic of the roll-to-roll coating setup. The pressure drop in the slot was controlled by adjusting the air pressure to the feed vessel. The wet coating thickness was controlled by precisely adjusting the gap between the substrate and the slot lips, and the excess coating was metered off and collected into the tray below. Four additional infrared dryers (2 kW each) and four hot air dryers (2 kW each) were installed on the machine to help with the drying process. The suspension was coated with a coating speed of 3 mmin^−1^ onto the two different substrates with 300 *μ*m wet coating thickness and in 1 and 2 layers. The graphite foil samples are called GF300 and GF600 for one and two layers of 300 *μ*m wet coating respectively, and the kraft paper samples are called KP300 and KP600 for one and two layers of 300 *μ*m wet coating respectively.

### Sample preparation

After coating the sample and substrate thickness were measured with a Mahr Millitast 1083 digital indicator. The median of five measurements was calculated and the sample thickness was subtracted with the substrate thickness in order to obtain the dry thickness of the coating. The electrode porosity was measured using Hg-intrusion porosimetry. The dry coat weight was calculated from the wet coating thickness and the solids content of the nanographite-nanocellulose suspension. The electrode-coated graphite foil was cut into 50 mm-wide and 100 mm-long samples with an electrode size of 50 mm × 50 mm (one half of the cut sample were coated). The electrode-coated separator paper were cut into an equal sized electrodes but with 50 mm-wide and 100 mm-long graphite foil pieces as current collectors.

**Figure 6:**
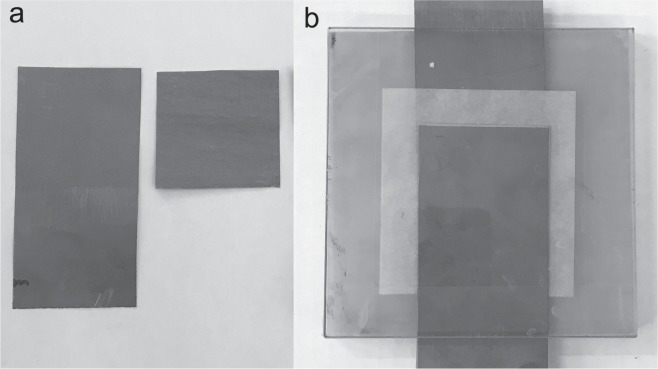
Photograph of; (**a**) electrode coated graphite foil (left) and electrode coated paper separator (right), (**b**) the assembled SC-device. The measuring equipment was connected to the protruding contacts of the SC assembly with four-point probes, where the voltage was measured on the same side of the current collector as the electrode was connected and the current was measured on the opposite side.

To assemble SC devices from GF300 and GF600, two electrode-coated graphite foils were put together, with the electrodes facing each other and with two 60 mm × 60 mm paper separators between them. For KP300 and KP600, two electrode-coated separator papers were put together between two 50 mm-wide and 100 mm-long graphite foil current collectors, with the electrodes facing the current collectors. In this way samples from both substrates were assembled to flat SCs with identical cell structure; : graphite foil - electrode - separator - separator - electrode - graphite foil. The SC devices were soaked in electrolyte for 5 min and then placed between two thick plastic plates as support, see Fig. 6. During measurement 1000 g of weight were put on top of the SC to ensure that all samples remained flat and experienced the same pressure.

Furthermore, cross-section images of the electrode-coated substrates were obtained using a field emission scanning electron microscope (TESCAN MAIA3-2016) at 5 kV. SEM images was obtained to analyze structural differences in the coatings due to the choice of substrate. The cross-section samples were cut with a IM400 Ion milling system from Hitachi and then coated with 5 nm of Iridium by sputtering.

### Electrical measurements

Three different electrical measurements were performed on the SCs; sheet resistance, CV and GCD. CV was performed on all SC units with a Versastat4 and scan rates of 10 mV/s, 50 mV/s and 100 mV/s. Using the same cell configuration, GCD were performed immediately after CV using a LabVIEW-based PXI system. The collected data were analyzed according to the method described by Stoller and Ruoff^28^. The specific capacitance, *C*_*sp*_, was calculated from the discharge curve in GCD measurements using equation (1) and (2), and the mean value of two identically prepared devices was determined.

1 $$C=I\cdot \frac{dt}{dV},$$

where *I* is the discharge current, *t* is the discharge time and *V* is the cell voltage. The discharge current *I* was set to a current density of 0.52 mA/cm^2^, 1.04 mA/cm^2^ and 2.08 mA/cm^2^, which corresponds to 0.5 A/g, 1 A/g and 2 A/g in relation to to the electrode weight of GF300 and KP300. Current per unit area was used to give a fair comparison in regard to ESR for the four different samples. The SCs were cycled for 10 cycles at each current density, and the capacitance and ESR were obtained from the 10th cycle. Further cycling for 2000 cycles were performed at 1.04 mA/cm^2^ to analyze the cycle stability (cyclability) in terms of ESR. The specific capacitance, *C*_*sp*_ was calculated by using Equation (2):

2 $${C}_{sp}=4\cdot \frac{C}{2\cdot m},$$

where *m* is the mass of one electrode. The ESR was calculated by dividing the resistive voltage drop, generated between charging and discharging, with the change in current. The sheet resistance was measured using a Keithley 2611A four-point-probe system. The mean value of 10 measurements was calculated.
